# An improved generalized Newton method for absolute value equations

**DOI:** 10.1186/s40064-016-2720-5

**Published:** 2016-07-11

**Authors:** Jingmei Feng, Sanyang Liu

**Affiliations:** School of Mathematics and Statistics, Xidian University, Xi’an, 710126 China; Department of Engineering Management, Shaanxi Radio and TV University, Xi’an, 710119 China

**Keywords:** Absolute value equations, Generalized Newton’s method, Global and local convergence

## Abstract

In this paper, we suggest and analyze an improved generalized Newton method for solving the NP-hard absolute value equations $Ax-|x|=b$ when the singular values of A exceed 1. We show that the global and local quadratic convergence of the proposed method. Numerical experiments show the efficiency of the method and the high accuracy of calculation.

## Background

We consider the absolute value equations (AVEs):

1 $$\begin{aligned} Ax-|x| = b, \end{aligned}$$

where $A\in R^{n\times n}$, $b\in R^n$ , and |*x*| denotes a vector in $R^n$, whose *i*-th component is $|x_i|$. A more general form of the AVEs, $Ax+B|x|=b$, was introduced by Rohn (2004) and researched in a more general context in Mangasarian (2007a). Hu et al. (2011) proposed a generalized Newton method for solving absolute value equation $Ax+B|x|=b$ associated with second order cones, and showed that the method is globally linearly and locally quadratically convergent under suitable assumptions. As was shown in Mangasarian and Meyer (2006) by Mangasarian, the general NP-hard linear complementarity problems (LCPs) (Cottle and Dantzing 1968; Chung 1989; Cottle et al. 1992) subsume many mathematical programming problems such as absolute value equations (AVEs) (1), which own much simpler structure than any LCP. Hence it has inspired many scholars to study AVEs. And in Mangasarian and Meyer (2006) the AVEs (1) was investigated in detail theoretically, the bilinear program and the generalized LCP were prescribed there for the special case when the singular values of *A* are not less than 1. Based on the LCP reformulation, sufficient conditions for the existence and nonexistence of solutions are given in this paper. Mangasarian also has used concave minimization model (Mangasarian 2007b), dual complementarity (Mangasarian 2013), linear complementarity (Mangasarian 2014a), linear programming (Mangasarian 2014b) and a hybrid algorithm (Mangasarian 2015) to solve AVEs (1). Hu and Huang reformulated a system of absolute value equations as a standard linear complementarity problem without any assumption and gave some existence and convexity results for the solution set of the AVEs (1) in Hu and Huang (2010). Paper Zhang et al. (2015) presented a new algorithm which relaxes the absolute value equations into a convex optimization problem, Zhang et al. found the sparsest solution of AVEs by the minimum $l_0$-norm. Caccetta et al. proposed a globally and quadratically convergent method for AVEs in Caccetta et al. (2011). Rohn et al. gave an iterative method for AVEs (1) and analyzed the sufficient conditions of unique solvability by Rohn et al. (2014), Uniqueness is always a hot spot (Wu and Guo 2015), and Moosaei et al. gave the minimum norm solution of absolute value equations $Ax-|x| = b$ which has multiple solutions (at most $2^n$) via simulated annealing algorithm in Moosaei et al. (2014). Salkuyeh (2014) put forward a hybrid algorithm which combined with skew-Hermitian and Picard-Hermitian splitting iteration method for solving AVEs (1), and gave the convergence analysis of the hybrid algorithm. Furthermore, Mangasarian (2009) clearly showed that generalized Newton method is a very effective method by solving some high dimensional examples in very few iterations. Haghani (2015) proposed an improved Newton method with two-step form, called Traub’s method, whose effectiveness is better than that of Mangasarian’s. Iqbal et al. (2015) proposed a Levenberg–Marquardt method for AVEs (1), which is the combination of steepest descent and the Gauss–Newton method. Paper Cruz et al. (2016) raised an inexact semi-smooth Newton algorithm for AVEs (1), and proved that the method is globally convergent. So, how to solve absolute value equations based on the classic Newton algorithm have been received many of the concerns. That have motivated us for trying to improve Newton method as the main aim of the present paper.

Now we describe our notation. The scalar product of two vectors *x* and *y* in the *n*-dimensional real space will be denoted by $\langle x,y\rangle$. For $x\in R^n$, the norm $\Vert x\Vert$ will denote the two-norm $(x^T x)^{1/2}$ , and ${ sign}(x)$ will denote a vector with components equal to $+1$, 0 or $-1$, depending on whether the corresponding component of *x* is positive, zero or negative, respectively. In addition, diag(sign(*x*)) will denote a diagonal matrix corresponding to sign (*x*).

In “Preliminary” section of the present work we give the notations and preliminary notes about AVEs. “The improved generalized Newton method” section gives an improved generalized Newton iterative algorithm which is globally and locally quadratic convergent under certain assumptions. In “Computational results” section some numerical reports show the efficiency of the proposed scheme. “Conclusions” section gives some concluding remarks to end the paper.

## Preliminary

We begin by defining the piece-wise linear vector function *f*(*x*) specified by the AVEs (1) as follows:

2 $$\begin{aligned} f(x)=Ax-|x|-b. \end{aligned}$$

A generalized Jacobian of *f* at *x* is

3 $$\begin{aligned} \partial f(x)=Ax-D(x), \end{aligned}$$

where $$D(x)=\partial |x| ={ diag}({ sign}(x))$$.

To solve (1), the iterative computational method of Mangasarian (2009) is as follows:

4 $$\begin{aligned} x^{k+1}=(A-D(x^k))^{-1}b, \quad k=0,1,2,\ldots . \end{aligned}$$

Another method proposed by Haghani (2015) is as follows:

5 $$\begin{aligned} y^k & = (A-D(x^k))^{-1}b,\nonumber \\ x^{k+1} & = {} y^k-(A-D(x^k))^{-1}((A-D(y^k))y^k-b), \quad k=0,1,2,\ldots . \end{aligned}$$

It has been shown that both the sequences $\{x^k\}$ generated by Eqs. (4) and (5) converge linearly to the true solution $\bar{x}$ of AVEs (1) when the singular values of *A* are exceed 1. However, the computational time of (5) is a little less than that of (4), with a higher residual error precision than (4).

## The improved generalized Newton method

Ostrowski (1960) and Traub (1964) presented a modified Newton’s iteration for solving nonlinear equation in real space *R*, which give us some inspiration. We will promote this idea to the *n*-dimensional space. The iterative method is as follows:

6 $$\begin{aligned} y^k & = {} x^k-(A-D(x^k))^{-1}f(x^k),\nonumber \\ a^k & = {} \frac{\Vert f(y^k)\Vert }{\Vert 2f(y^k)-f(x^k)\Vert },\nonumber \\ x^{k+1} & = {} y^k-a^k(y^k-x^k), \quad k=0,1,2,\ldots . \end{aligned}$$

We can simplify (6) as much as possible to obtain the follwing form of improved generalized Newton method for AVEs

7 $$\begin{aligned} x^{k+1}=x^k+(1-a^k)d^k, \quad k=0,1,2,\ldots , \end{aligned}$$

where $d^k:=-(A-D(x^k))^{-1}(Ax^k-|x^k|-b)$. It is clear that our method is a Newton method with a specific liner search.

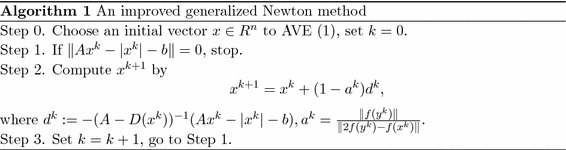



We shall need a few theoretical results to establish convergence of Algorithm 1, we first quote the following two results from Mangasarian and Meyer (2006).

### **Lemma 1**

*The singular values of the matrix*$A\in R^{n\times n}$*exceed 1 if and only if the minimum eigenvalue of*$A'A$*exceeds 1*.

### **Lemma 2**

*If the singular values of*$A\in R^{n\times n}$*exceed 1 for the method* (6), *then*$(A-D)^{-1}$*exists for any diagonal matrixDwhose diagonal elements are*$\pm 1$*or* 0.

Therefore, the sequence of vector iterates form (6) is well defined no matter how to choose the initial vector $x^0$ in $R^n$.

We now prove the proposed Newton direction $d^k$ of (7) is a descent direction for the objective function $\Vert f(x)\Vert ^2$.

### **Lemma 3**

*If the singular values of*$A\in R^{n\times n}$*exceed 1, then the proposed Newton direction*$d^k$*of* (7) *is a descent direction for the objective function*$F(x)=\frac{1}{2}\Vert f(x)\Vert ^2$.

### *Proof*

Since $f(x)=Ax-|x|-b, \partial f(x)=A-D(x)$, and $(A-D(x))^{-1}$ exists for any diagonal matrix *D* whose diagonal elements are $\pm 1$ or 0.

In addition, we know

$$\begin{aligned} d^k=-(\partial f(x))^{-1}f(x)=-(A-D(x^k))^{-1}(Ax^k-|x^k|-b). \end{aligned}$$

and

$$\begin{aligned} x^{k+1} & = {} y^k-a^k(y^k-x^k)\\ & = {} (1-a^k)y^k+a^kx^k\\ & = {} (1-a^k)(x^k+d^k)+a^kx^k\\ & = {} x^k+(1-a^k)d^k. \end{aligned}$$

Moreover, $$F(x)=\frac{1}{2}\Vert f(x)\Vert ^2$$, then $$\partial F(x)=\partial f(x)f(x)$$.

So

$$\begin{aligned} \langle \partial F(x),d^k\rangle & = {} \langle \partial f(x)f(x),-(\partial f(x))^{-1}f(x)\rangle \\ & = {} -(f(x))^{T}(\partial f(x))^{T}(\partial f(x))^{-1}f(x)\\ & = {} -\Vert f(x)\Vert ^2<0. \end{aligned}$$

Consequently, $d^k$ is a descent direction of *F*(*x*). $\square$

### **Lemma 4**

*Let the singular values ofAexceed 1, then the sequence*$\{x_k\}$*generated by the improved generalized Newton method* (6) *is bounded, and there exists an accumulation point*$\bar{x}$*such that*$(A-\bar{D})\bar{x}=b+\bar{a} f(\bar{x})$*for some diagonal matrixes*$\bar{D}$*with diagonal elements of*$\pm 1$*or* 0.

### *Proof*

Suppose that the sequence $\{x^k\}^\infty _{k=0}$ is unbounded. Then there exists a subsequence $\{x^{k_j+1}\}\rightarrow \infty$ with nonzero $x^{k_j+1}$, such that $D(x^{k_j})=\widetilde{D}$, where $\widetilde{D}$ is a assured diagonal matrix with diagonal elements equal to $\pm 1$ or 0 extracted from the finite number of possible configurations for $D(x^k)$ in the sequence $\{D(x^k)\}$, and such that the bounded subsequence $\left\{ \frac{x^{k_j+1}}{\Vert x^{k_j+1}\Vert }\right\}$ converges to $\widetilde{x}$. By Eq. (6), we have

$$\begin{aligned} y^k & = {} x^k-(A-D(x^k))^{-1}f(x^k)\\ & = {} x^k-(A-D(x^k))^{-1}(Ax^k-|x^k|-b)\\ & = {} x^k-(A-D(x^k))^{-1}((A-D(x^k))x^k-b)\\ & = {} x^k-(A-D(x^k))^{-1}(A-D(x^k))x^k+(A-D(x^k))^{-1}b\\ & = {} x^k-x^k+(A-D(x^k))^{-1}b\\ & = {} (A-D(x^k))^{-1}b.\\ x^{k+1} & = {} y^k-a^k(y^k-x^k)\\ & = {} (A-D(x^k))^{-1}b+a^k(A-D(x^k))^{-1}f(x^k)\\ & = {} (A-D(x^k))^{-1}(b+a^kf(x^k)). \end{aligned}$$

So, $$(A-D(x^k))x^{k+1}=b+a^kf(x^k)$$, thus, $$(A-D(x^{k_j}))\frac{x^{k_{j+1}}}{\Vert x^{k_{j+1}}\Vert }=\frac{b+a^{k_j}f(x^{k_j})}{\Vert x^{k_{j+1}}\Vert }$$.

Due to the following equation:

8 $$\begin{aligned} \Vert a^{k_j}\cdot f(x^{k_j})\Vert =\frac{\Vert f(y^{k_j})\Vert \cdot \Vert f(x^{k_j})\Vert }{\Vert 2f(y^{k_j})-f(x^{k_j})\Vert }, \end{aligned}$$

and Lemma 3, the Newton direction $d^k$ in (7) is a descent direction for the objective function $\Vert f(x)\Vert ^2$. We get $\Vert a^{k_j}\cdot f(x^{k_j})\Vert \rightarrow 0$, as $j\rightarrow \infty$.

Now, $j\rightarrow \infty$ gives us:

$$\begin{aligned} (A-\widetilde{D})\widetilde{x}=0, \quad \Vert \widetilde{x}\Vert =1, \end{aligned}$$

since $x^{k_j+1}\rightarrow \infty$. This is a contradiction with the nonsingularity of $(A-D)$ which follows from Lemma 2. Hence, the vector sequence $\{x^k\}$ is bounded and there exists an accumulation point $(\bar{D},\bar{x})$ of $\{(D(x^k),x^{k+1})\}$ such that

$$\begin{aligned} \bar{x}=(A-\bar{D})^{-1}(b+\bar{a} f(\bar{x})). \end{aligned}$$

The proof is complete. $\square$

### **Theorem 1**

(Global linear convergence) *If*$\Vert (A-D)^{-1}\Vert <\frac{1}{3}$*for any diagonal matrixDwith diagonal elements of*$\pm 1$*or* 0, *then the improved generalized Newton method* (6) *converges linearly from any starting point*$x^0$*to a solution*$\bar{x}$*for any solvable AVEs* (1).

### *Proof*

Suppose that $\bar{x}$ is a solution of the AVE (1). Noting that $D(\bar{x}) \bar{x}=|\bar{x}|$ and $D( x^k) x^k=|x^k|$, for convenience, let $\bar{D}=D(\bar{x})$, $D^k=D(x^k)$. Subtracting $(A-D(\bar{x}))\bar{x}=b$ from $(A-D(x^k))x^{k+1}=b+a^kf(x^k)$, we get

$$\begin{aligned} A(x^{k+1}-\bar{x}) & = {} D^k x^{k+1}-\bar{D} \bar{x}+a^kf(x^k)\\ & = {} D^k(x^{k+1}-x^k+x^k)-\bar{D} \bar{x}+a^kf(x^k) \\ & = {} |x^k|-|\bar{x}|+D^k(x^{k+1}-x^k)+a^kf(x^k)\\ & = {} |x^k|-|\bar{x}|+D^k(x^{k+1}-\bar{x}+\bar{x}-x^k)+a^kf(x^k)\\ & = {} |x^k|-|\bar{x}|+D^k(x^{k+1}-\bar{x})+D^k(\bar{x}-x^k)+a^kf(x^k). \end{aligned}$$

So,

$$\begin{aligned} (A-D^k)(x^{k+1}-\bar{x})=|x^k|-|\bar{x}|+D^k(\bar{x}-x^k)+a^kf(x^k). \end{aligned}$$

i.e.,

$$\begin{aligned} (x^{k+1}-\bar{x}) =(A-D^k)^{-1}(|x^k|-|\bar{x}|+D^k(\bar{x}-x^k)+a^kf(x^k)). \end{aligned}$$

From Mangasarian (2009, Lemma 5), we know that for $\forall x, y \in R^n,\Vert |x|-|y|\Vert \le 2\Vert x-y\Vert$.

Thus,

$$\begin{aligned} \Vert x^{k+1}-\bar{x}\Vert & = {} \Vert (A-D^k)^{-1}(|x^k|-|\bar{x}|+D^k(\bar{x}-x^k)+a^kf(x^k))\Vert \\ & \le {} \Vert (A-D^k)^{-1}\Vert (\Vert |x^k|-|\bar{x}|\Vert +\Vert D^k(\bar{x}-x^k)\Vert +\Vert a^kf(x^k))\Vert )\\ & \le {} \Vert (A-D^k)^{-1}\Vert (2\Vert x^k-\bar{x}\Vert +\Vert \bar{x}-x^k\Vert +\Vert a^kf(x^k))\Vert ) \\ & \le {} \Vert (A-D^k)^{-1}\Vert (3\Vert x^k-\bar{x}\Vert +\Vert a^kf(x^k))\Vert )\\ & < {} \Vert x^k-\bar{x}\Vert +\frac{1}{3}\Vert a^kf(x^k))\Vert . \end{aligned}$$

Since, $\Vert a^{k}\cdot f(x^{k})\Vert \rightarrow 0$, as $k\rightarrow \infty$, so the sequence$\{\Vert x^k-\bar{x}\Vert \} \rightarrow 0$, as $k \rightarrow \infty$. Consequently, $\{x^k\}$ converges to $\bar{x}$.

The proof is complete. $\square$

### **Theorem 2**

*If*$\Vert A^{-1}\Vert <\frac{1}{4}$*and*$D(x^k)\ne 0$*for any diagonal matrix*$D(x^k)$*with diagonal elements of*$\pm 1$*or* 0, *then the Algorithm 1 converges linearly from any starting point*$x^0$*to a solution*$\bar{x}$*for any solvable AVEs* (1).

### *Proof*

The proof directly from Mangasarian (2009, Proposition 7). It is hence omitted.

In the following, we use Eq. (7) to prove the locally quadratic convergence of the Algorithm 1. $\square$

### **Lemma 5**

*If*$A-D$*is nonsingular for any diagonal matrixDwith diagonal elements of*$\pm 1$*or* 0, *then the Algorithm 1 is approximately Newton’s method*.

### *Proof*

Taking into account the step length

$$\begin{aligned} 1-a^k=\frac{\Vert f(y^k)-f(x^k)+f(y^k)\Vert -\Vert f(y^k)\Vert }{\Vert 2f(y^k-f(x^k))\Vert }\le \frac{\Vert f(y^k)-f(x^k)\Vert }{\Vert 2f(y^k)-f(x^k)\Vert }. \end{aligned}$$

When $x^k$ is in a neighborhood of the solution $\bar{x}$ of AVEs (1), $f(y^k)$ is close to $f(x^k)$, we perform a Newton step from $x^k$ to $y^k$, for larger *k*, $f(y^k) =O(\Vert y^k-\bar{x}\Vert ^2)$, thus

$$\begin{aligned} f(y^k)-f(x^k) & = {} Ad^k+|x^k|-|x^k+d^k| \\ & = {} Ad^k-D(x^k)d^k+O(\Vert d^k\Vert ^2) \\ & = {} -f(x^k)+O(\Vert d^k\Vert ^2). \end{aligned}$$

So, $2f(y^k)-f(x^k)=f(y^k)-f(x^k)+O(\Vert d^k\Vert ^2)$, when *k* is sufficiently large and $(A-D(x^k)$ is uniformly bounded $(|A-D(x^k)|<|A|+|D(x^k)|<|A|+1)$, $d^k=O(f(x^k))$. Then, $f(y^k)-f(x^k)=-f(x^k)+O(\Vert f(x^k)\Vert ^2)$, we also have $\Vert f(x^k)\Vert =\Vert (A-D(\overline{}\overline{x}))(x^k-\overline{x})\Vert =O(\Vert x^k-\overline{x}\Vert )=:O(\Vert c^k\Vert )$, where $c^k:=x^k-\overline{x}$. Thus, $f(y^k)=O(\Vert c^k\Vert ^2)$, and

$$\begin{aligned} 1-a^k=1-\frac{O(\Vert c^k\Vert ^2)}{O(\Vert c^k\Vert )}=1-O(\Vert c^k\Vert ). \end{aligned}$$

Hence, our method is approximately the generalized Newton’s method. $\square$

### **Theorem 3**

(Locally quadratic convergence) *If*$A-D$*is nonsingular for any diagonal matrixDwith diagonal elements of*$\pm 1$*or* 0, *then the sequence*$\{x^k\}$*from improved generalized Newton’s method* (7) *converges to*$\bar{x}$*and*$\Vert x^{k+1}-\bar{x}\Vert =O(\Vert x^k-\bar{x}\Vert ^2)$.

### *Proof*

This theorem can be proved in a similar way as Theorem 2 by Qi and Sun (1993). We omit it here. $\square$

## Computational results

To illustrate the implementation and efficiency of the proposed method, we test the following two examples. All the experiments are performed by MATLAB R2010a. In comparisons, NM, TM and INM denote the generalized Newton’s method (4), the generalized Traub’s method (5) and the improved generalized Newton’s method (6), respectively.

### *Example 1*

We choose randomly matrix *A* according to the following formula:

$$\begin{aligned} A=rand(n*(eye(n,n)-0.02*(2*rand(n,n)-1))). \end{aligned}$$

Then, we choose a random $x\in R^n$, compute $b=Ax-|x|$, and denote the problem size by *n*. To ensure that the minimum singular value of each A exceeds 1, we compute the minimum singular value of *A* and reseal *A* by the minimum singular value multiply by a random number in the interval [1, 2], The results are shown in Table 1.

**Table 1:** The comparison of NM, TM and INM in Example 1

Dim	NM			TM			INM		
	K	ACC	T	K	ACC	T	K	ACC	T
100	3	$1.6125\times 10^{-10}$	0.0036	3	$1.8416\times 10^{-11}$	0.0031	3	$1.6168\times 10^{-11}$	0.0027
200	3	$6.0215\times 10^{-10}$	0.0097	3	$7.0496\times 10^{-11}$	0.0095	3	$6.2749\times 10^{-11}$	0.0084
300	3	$1.2931\times 10^{-9}$	0.0216	3	$1.6284\times 10^{-10}$	0.0071	3	$4.5510\times 10^{-10}$	0.0217
400	3	$2.5705\times 10^{-9}$	0.0567	3	$2.6186\times 10^{-10}$	0.0517	3	$7.6406\times 10^{-10}$	0.0321
500	3	$4.3078\times 10^{-8}$	0.0831	3	$4.7102\times 10^{-10}$	0.0944	3	$3.5408\times 10^{-10}$	0.0512
600	4	$6.3700\times 10^{-8}$	0.1365	3	$5.5877\times 10^{-10}$	0.1576	3	$4.1326\times 10^{-10}$	0.0906
700	4	$7.0085\times 10^{-8}$	0.2227	3	$1.0945\times 10^{-9}$	0.2304	3	$8.8369\times 10^{-10}$	0.1278
800	4	$2.1838\times 10^{-7}$	0.3766	4	$2.0634\times 10^{-9}$	0.3103	4	$2.5631\times 10^{-9}$	0.1788
900	4	$3.6958\times 10^{-7}$	0.4706	4	$6.9784\times 10^{-9}$	0.4173	4	$2.9899\times 10^{-9}$	0.2325
1000	5	$6.3484\times 10^{-7}$	0.6973	4	$1.7392\times 10^{-8}$	0.5221	4	$6.3255\times 10^{-9}$	0.3063

### *Example 2*

The matrix *A* of which all the singular values are greater than 1 is generated by the following MATLAB procedure:

$$\begin{aligned} rand(\text{`}state\text{'},0); R=rand(n,n); A=R'*R+n*eye(n); b=(A-eye(n,n))*ones(n,1). \end{aligned}$$

And, the results are shown in Table 2.

**Table 2:** The comparison of NM, TM and INM in Example 2

Dim	NM			TM			INM		
	K	ACC	T	K	ACC	T	K	ACC	T
100	3	$1.7851\times 10^{-11}$	0.0042	3	$1.1360\times 10^{-11}$	0.0333	3	$1.2528\times 10^{-11}$	0.0029
200	3	$3.3603\times 10^{-10}$	0.0146	3	$8.0510\times 10^{-11}$	0.0132	3	$6.7278\times 10^{-11}$	0.0069
300	3	$3.9958\times 10^{-9}$	0.0392	3	$2.8067\times 10^{-10}$	0.0301	3	$2.6026\times 10^{-10}$	0.0203
400	3	$7.3587\times 10^{-9}$	0.0857	3	$6.8738\times 10^{-10}$	0.0681	3	$5.7847\times 10^{-10}$	0.0444
500	3	$2.1626\times 10^{-9}$	0.1411	3	$1.1259\times 10^{-9}$	0.1085	3	$1.1792\times 10^{-9}$	0.0679
600	4	$1.1403\times 10^{-8}$	0.2356	3	$2.1310\times 10^{-9}$	0.1595	3	$2.4023\times 10^{-9}$	0.1055
700	4	$4.6354\times 10^{-8}$	0.3993	3	$2.9880\times 10^{-9}$	0.2681	3	$3.6051\times 10^{-9}$	0.1704
800	4	$5.7742\times 10^{-8}$	0.4829	4	$5.1658\times 10^{-9}$	0.3543	3	$3.8475\times 10^{-9}$	0.2239
900	5	$6.4563\times 10^{-8}$	0.5749	4	$6.9541\times 10^{-9}$	0.4665	3	$7.0228\times 10^{-9}$	0.3076
1000	5	$8.6322\times 10^{-8}$	0.7589	4	$8.8558\times 10^{-9}$	0.6615	3	$7.3382\times 10^{-9}$	0.4127

In Tables 1 and 2, Dim, K, ACC and T denote the dimensions of the problem, the number of iterations, $\Vert Ax^k-|x^k|-b\Vert _2$ and times(s), respectively. It is evident from Tables 1 and 2 that the improved generalized Newton’s method is very effective for solving large problems.

**Fig. 1:**
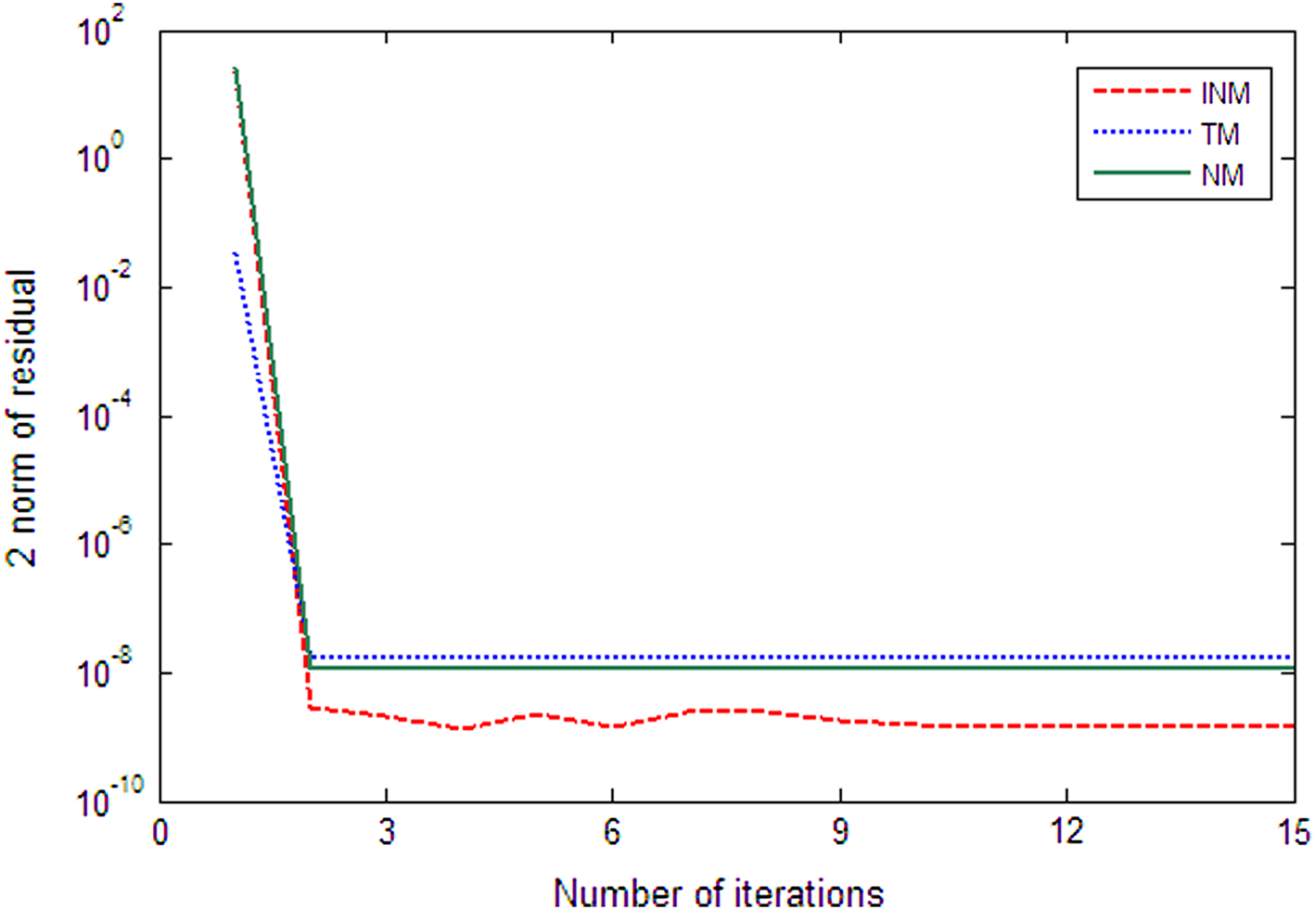
Comparison of NM, TM and INM for Example 1 with $n=1000$

**Fig. 2:**
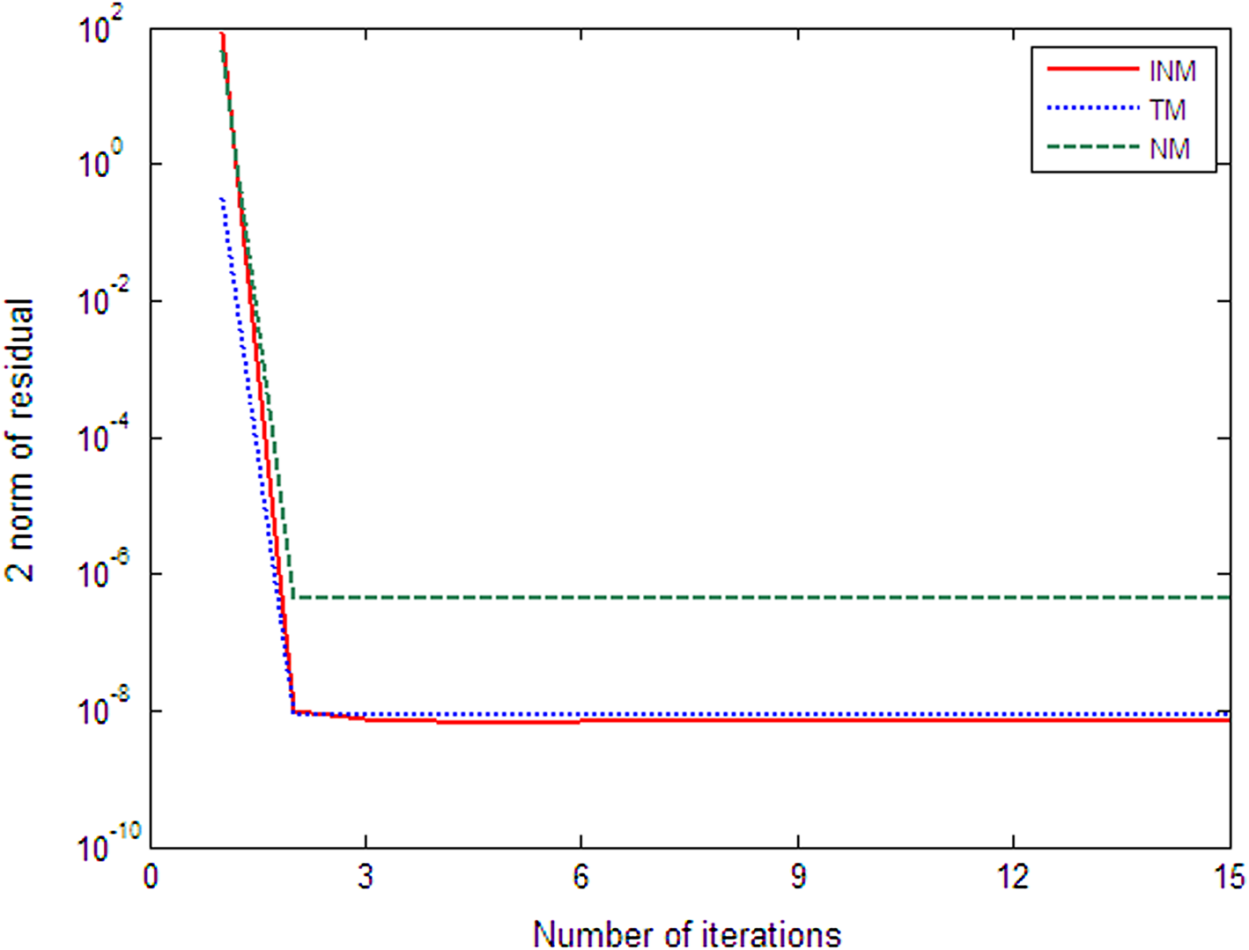
Comparison of NM, TM and INM for Example 2 with $n=1000$

We give below the convergence curves of three algorithms for solving Examples 1, 2 by Figs. 1 and 2. We can see that the convergence of the INM is better than NM’s and TM’s.

## Conclusions

In this paper, we have proposed the generalized Newton’s method with special search direction for solving the NP-hard absolute value equations under certain assumptions on *A*. The method have some nice convergence properties and calculation results. Further work is to find more effective methods for AVEs.
